# Placing Cognitive Rigidity in Interpersonal Context in Psychosis: Relationship With Low Cognitive Reserve and High Self-Certainty

**DOI:** 10.3389/fpsyt.2020.594840

**Published:** 2020-11-26

**Authors:** Helena García-Mieres, Judith Usall, Guillem Feixas, Susana Ochoa

**Affiliations:** ^1^Parc Sanitari Sant Joan de Déu, Barcelona, Spain; ^2^Mental Health Networking Biomedical Research Center, CIBERSAM, Madrid, Spain; ^3^Department of Clinical Psychology and Psychobiology, Faculty of Psychology, University of Barcelona, Barcelona, Spain; ^4^The Institute of Neurosciences, University of Barcelona, Barcelona, Spain

**Keywords:** self, schizophrenia, repertory grid, personal construct psychology, dichotomous thinking, cognitive bias

## Abstract

**Introduction:** People with psychosis show impairments in cognitive flexibility, a phenomenon that is still poorly understood. In this study, we tested if there were differences in cognitive and metacognitive processes related to rigidity in patients with psychosis. We compared individuals with dichotomous interpersonal thinking and those with flexible interpersonal thinking.

**Methods:** We performed a secondary analysis using two groups with psychosis, one with low levels of dichotomous interpersonal thinking (*n* = 42) and the other with high levels of dichotomous interpersonal thinking (*n* = 43). The patients were classified by splitting interpersonal dichotomous thinking (measured using the repertory grid technique) to the median. The groups were administered a sociodemographic questionnaire, a semi-structured interview to assess psychotic symptoms [Positive and Negative Syndrome Scale (PANSS)], a self-report of cognitive insight [Beck Cognitive Insight Scale (BCIS)], neurocognitive tasks [Wisconsin Card Sorting Test (WCST) and Wechsler Adult Intelligence Scale (WAIS)], and the repertory grid technique. We used a logistic regression model to test which factors best differentiate the two groups.

**Results:** The group with high dichotomous interpersonal thinking had earlier age at onset of the psychotic disorder, higher self-certainty, impaired executive functioning, affected abstract thinking, and lower estimated cognitive reserve than the group with flexible thinking. According to the logistic regression model, estimated cognitive reserve and self-certainty were the variables that better differentiated between the two groups.

**Conclusion:** Cognitive rigidity may be a generalized bias that affects not only neurocognitive and metacognitive processes but also the sense of self and significant others. Patients with more dichotomous interpersonal thinking might benefit from interventions that target this cognitive bias on an integrative way and that is adapted to their general level of cognitive abilities.

## Introduction

People with psychosis exhibit impairments in cognitive flexibility (1, 2), a phenomenon considered a fundamental aspect of health with a major contribution on daily well-being. Cognitive flexibility refers to several dynamic processes that unfold over time and is reflected in how a person adapts to fluctuating situational demands, reconfigures mental resources, or shifts perspective (3). In psychosis, cognitive flexibility has been defined from two main approaches and using a variety of metacognitive and neurocognitive measures. As a metacognitive process, it is a complex higher order reasoning construct. It includes an individual's ability to release from a strongly held belief, once formed, in order to engage in further cognitive operations involved in making judgments under conditions of uncertainty: rethinking the possibility of being mistaken; reviewing the main belief in light of newer evidence/information; and generating and considering other explanations (4). In contrast, as a neurocognitive process, it is considered a component of executive functioning. In this sense, cognitive flexibility refers to the ability to switch thought and/or response patterns and target-directed behaviors. Further, cognitive flexibility is critical in using feedback to modify cognitive sets. Essentially, in the context of neurocognition, the paradigm has referred to the inability to set-shifting, also called “stuck-in-set behavior” (1).

From the metacognitive approach, impairments in cognitive flexibility in psychosis, also termed in the literature as belief inflexibility, have been mainly discussed in the context of reasoning about clinical delusions (4–6). People with psychosis exhibit impaired cognitive flexibility when reflecting about their delusional beliefs. One form of this cognitive rigidity is the construct of self-certainty (7), which suggests that the individual is excessively convinced of the accuracy of their own beliefs and is resistant to change their ideas. Individuals with psychosis are often overconfident in errors that maintain delusional beliefs, thus resulting in difficulties appreciating that one may be mistaken and refusing alternative explanations (2, 8). This reasoning process is altered in psychosis as compared with non-psychiatric controls (9) and is a predictor of treatment response (10).

From the neurocognitive approach, the relative inability to shift attentional set became the paradigm case of a cognitive consequence of frontal lobe alterations, based on the results of early studies using the Wisconsin Card Sorting Test (WCST) (1, 11, 12). Cognitive rigidity in psychosis has been largely studied using the WCST. Patients make more perseverative errors and complete a smaller number of categories than healthy controls (11). However, this pattern of results is not specific to psychotic disorders (13), and impairments in performing this task may strongly rely on general intellectual abilities (2). Waltz suggests that excessive cognitive rigidity is likely to be characteristic of subgroups of patients with specific disorder profiles (3, 12). This idea is supported by empirical studies that have detected subgroups of patients with different performance on the WCST (14–16). For instance, patients with a general and marked executive functioning impairment showed lower IQ and severe negative symptomatology. Accordingly, identifying the characterization of subgroups of patients with psychosis suffering cognitive rigidity may be of interest in current research.

Understanding the complexity of cognitive rigidity in psychosis may benefit from a wider conceptualization, such as the one provided by the personal construct theory (PCT). According to PCT (17, 18), people construe the self and others using a system of personal constructs, which form a complex and hierarchical network. Personal constructs are bipolar dimensions of meaning, which are constructed by the individual. People use this system to define and interpret their self and the people who constitute their main interpersonal world. When this system is rigid, it can manifest as a pattern of dichotomous interpersonal thinking. The dimension of dichotomous interpersonal thinking (polarized thinking) when interpreting the self and significant others reflects a thinking tendency to understand oneself and the others in extreme or dichotomous terms (19). People with psychosis exhibit high dichotomous interpersonal thinking as compared with controls (20–22). This is relevant because high levels of it have been linked to more severity of positive symptoms (23), to more psychopathology in general (20), and to lower social functioning (24).

The relationship between dichotomous interpersonal thinking and other known processes of cognitive rigidity in metacognition and neurocognition in psychosis should be unraveled. Cognitive biases may intrinsically happen in the context of the construal of self and interpersonal relationships, thus possibly being more mobilizing and effective for outcomes in therapy (23, 25, 26). Moreover, the relationship between metacognitive and neurocognitive processes related to flexibility is still poorly understood and is considered an underdeveloped area of research (2). Therefore, deeper understanding of cognitive flexibility processes in psychosis is needed in current research to better target this outcome in therapy. Cognitive flexibility is a mediating factor in improving symptomatology in cognitive and metacognitive therapies for psychosis (27, 28). While it may also be subject to change (29–31), it is resistant to change by antipsychotic medication (32). Clarifying the facets of cognitive flexibility in psychosis and identifying different profiles of impairment may aid in developing tailored cognitive therapy programs but also to partially explain heterogeneity in psychosis.

### Objective and Hypothesis

We aimed to identify differences in cognitive and metacognitive processes in patients with psychosis. For this aim, we compared individuals with high and low dichotomous interpersonal thinking while controlling for symptomatic and sociodemographic factors. This procedure allowed us to gain a full picture of dichotomous interpersonal thinking in the context of other dimensions of cognitive rigidity in psychosis.

We hypothesized that cognitive rigidity may be a general and underlying cognitive bias in psychosis that involves many cognitive processes beyond reasoning about delusions, overconfidence on own beliefs (i.e., self-certainty), and neurocognitive processes related to flexibility (executive functioning) or affects the sense of self. In other words, patients with high dichotomous interpersonal thinking may have impairments in metacognitive and neurocognitive processes related to rigidity. Moreover, these variables will differentiate two profiles of patients. This hypothesis stems from previous literature that found associations between these cognitive processes. For instance, an association of poor executive functioning and global cognitive capacity with high self-certainty has been reported (6). Also, making over-confident decisions has been largely reported in people with schizophrenia (33–35). Other approaches to the sense of self in psychosis have also found that interpersonal self-concepts seem to be hampered when neurocognitive impairments occur (36). On previous work, we also found an association of high self-certainty with dichotomous interpersonal thinking with the same sample of the present study (23).

## Methods

### Participants and Procedure

A total of 85 outpatients with a confirmed diagnosis of a schizophrenia spectrum or related disorder were recruited from four participating mental health centers at Barcelona (Spain) and its surrounding area. As inclusion criteria, patients needed to have a diagnosis of schizophrenia, psychotic disorder not otherwise specified, delusional disorder, schizoaffective disorder, brief psychotic disorder, or schizophreniform disorder [according to the *Diagnostic and Statistical Manual of Mental Disorders* (Third Edition) (DSM-5)]; to be aged between 18 and 60 years; and to be clinically stable enough to do the interviews. Patients were excluded if they had an established diagnosis of traumatic brain injury, dementia, or intellectual disability (premorbid IQ <70); current substance dependence; or were hospitalized. This research is a secondary analysis from a study about the role of personal identity in psychosis (23). The sample had a heterogeneous profile in terms of diagnosis (45.9% of schizophrenia, 24.7% of schizoaffective disorder, 18.8–5% of psychosis not otherwise specified, 4.7% of schizophreniform disorder, 3.5% of brief psychotic disorder, and 2.4% of delusional disorder) and disorder chronicity (69.4% of prolonged psychosis and 30.6% of early psychosis). More details of the sample characteristics can be consulted in the primary study (23).

The clinicians of the participating mental health centers referred the participants that met the inclusion criteria and verbally agreed to participate in the study. The first author carried out all the assessments. After receipt of more exhaustive information about the study and signing the informed consent and after confirmation of inclusion criteria was made, a demographic questionnaire and the repertory grid technique (RGT) were administered in the first and second sessions, while a third session was used for the other instruments. The study was approved by the research ethics committee of the coordinating center (*Parc Sanitari Sant Joan de Déu*).

### Instruments

#### The Repertory Grid Technique (37–39)

Dichotomous interpersonal thinking was measured with the RGT, a semi-structured interview derived from the PCT. The RGT can adopt different flexible formats according to the aim of the study. In this case, we used an idiographic and interpersonal design, which assessed the personal meanings involved in personal identity, operationalized in terms of personal constructs. RGT is idiographic because personal constructs (i.e., the *items* of this instrument) are elicited from the participant rather than provided by the researcher, and it is interpersonal because these constructs are applied to a set of elements that represent other people who are relevant for the interviewee (parents, siblings, relatives, partners, and friends) evaluated along with “self now,” “ideal self,” and a “non-grata person” (someone they do not like). The dyadic method (34, 35) was used to elicit constructs, by comparing pairs of the mentioned elements and asking for differences and similarities between them (e.g., “nervous–calm”). After the elicitation procedure, participants rated each element of their grid on a 7-point Likert-type scale according to each construct elicited in the interview. An example of a repertory grid from one of our participants appears in a published case study (40). For the current study, we used the index of polarization (% of extreme ratings, “1” and “7” scores, in the grid data matrix) as a measure of dichotomous interpersonal thinking. This is considered a measure of dichotomous or extreme thinking in the interpersonal domain, a form of cognitive rigidity (19). High scores represent extremity, with the person having a tendency toward a dichotomous thinking style, while low scores are an indicator of flexible thinking.

#### The Positive and Negative Syndrome Scale

This scale was used to assess psychotic symptoms (41, 42). We used Wallwork's factor analysis to derive positive, excitative, and cognitive symptoms (43). The positive factor included four items: delusions, hallucinations, grandiosity, and unusual thought content. The excitative factor contained four items: excitement, hostility, uncooperativeness, and poor impulse control. The cognitive factor included three items: conceptual disorganization, difficulty in abstract thinking, and poor attention. In addition, we analyzed separately expressive and experiential deficits as negative symptoms subdomains following the factorial division by Khan et al. (44). The expressive factor contained blunted affect, poor rapport, lack of spontaneity, and motor retardation, while the experiential factor included emotional withdrawal, passive social withdrawal, and active social avoidance.

### Metacognition

#### The Beck Cognitive Insight Scale (7, 45)

This is a self-reported scale to measure cognitive insight. The Beck Cognitive Insight Scale is composed of two subscales: self-reflectivity and self-certainty. Both subscales are analyzed separately.

### Neurocognition

#### The Wisconsin Card Sorting Test (46)

This neuropsychological task was used in its abbreviated and computer version to measure executive function, set-shifting, and cognitive flexibility. We included the normative scoring of the indexes of total number of correct categories and perseverative errors.

#### The Vocabulary and Similarities Subtests of the Wechsler Adult Intelligence Scale (47)

The vocabulary subtest was used to measure premorbid IQ, an estimator used in research to assess cognitive reserve (48). The similarities subtest was used to assess ability for abstract thinking. The normative scoring was used in both cases.

### Data Analyses

We computed personal identity measures of data obtained from participants' repertory grids using GRIDCOR v4.0 (38) and entered these results in a database along with other measures.

**Figure 1:**
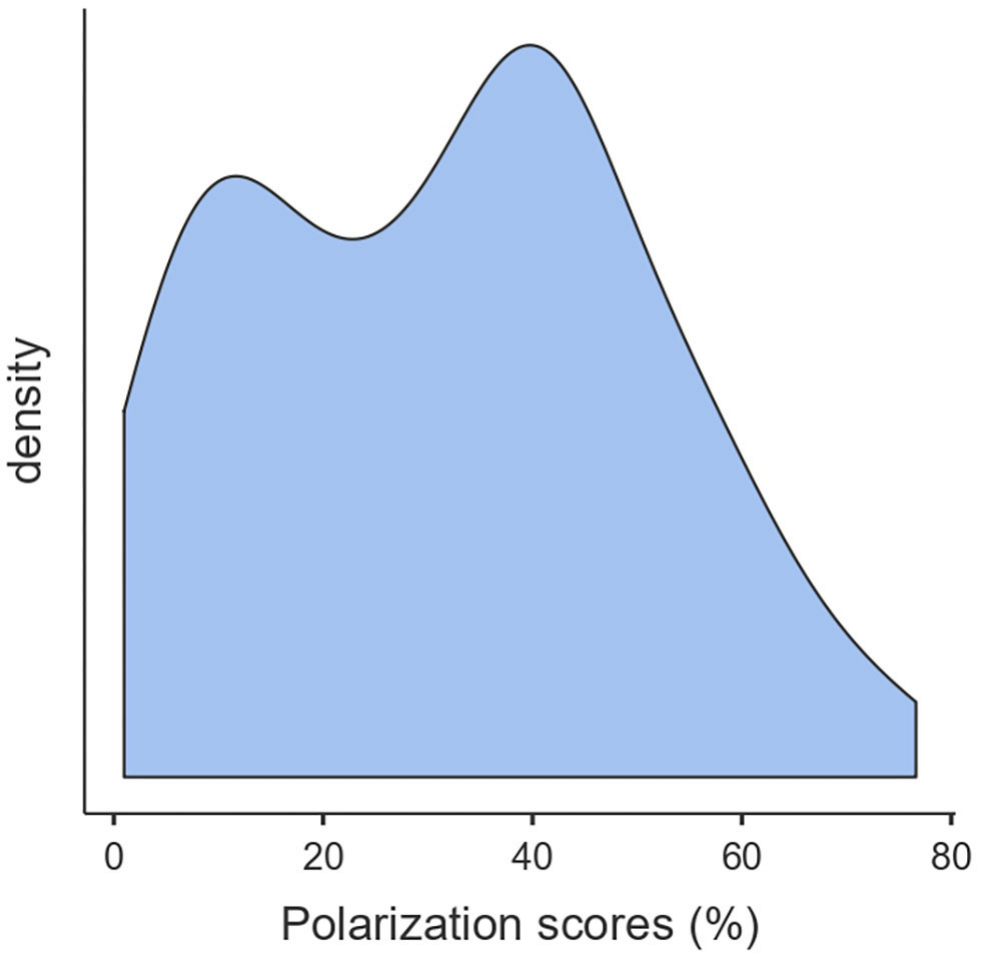
Distribution of the scores of the polarization index (dichotomous interpersonal thinking).

We conducted the main analyses in three stages. First, we tested the normal distribution of variables. Since the polarization index did not adjust to a normal distribution, after visual inspection of the distribution, we identified two distant modes. See Figure 1 for the bimodal distribution of scores of the polarization index. The descriptive analysis supported the identification of two modes on this index, one identified at the score of 11% and another one at the score of 41%. Therefore, we decided to divide the sample according to two groups, which were split according to the median of the distribution of scores in this index: high-dichotomous interpersonal thinking (polarization score over 33, meaning more than 33% of extreme scores in their grid) and low-dichotomous interpersonal thinking (polarization score equal or below to 33). Second, to better characterize these two groups, we performed a descriptive and comparative analysis using mean differences or chi-square tests for categorical variables. Third, we built a hierarchical binary logistic regression to detect the variables that were best able to differentiate between high- and low-polarized patients. We included as independent variables those that were significant at level *p* <0.05 in the bivariate analysis after demographic variables that were significant in the bivariate comparisons. On a second block, we added our measure of metacognition, the index of self-certainty, based on a previous analysis of this sample as it was already known to be associated with dichotomous interpersonal thinking (23). On following blocks, we included the neurocognitive measures that, based on theoretical consideration and the magnitude of the effect sizes found in the previous bivariate comparison, should be able to differentiate between patients with low and high levels of dichotomous interpersonal thinking (i.e., cognitive reserve-premorbid IQ on third block, abilities for abstract thinking on fourth block, and executive functioning on fifth block). On the last block, we included symptomatology factors that reached statistical significance on the bivariate comparisons. Effect sizes and their confidence intervals were also calculated. All the analyses were done using the jamovi 1.0 software (49).

## Results

Table 1 shows descriptive and comparative results for the sociodemographic, clinical, and cognitive variables for the high and low dichotomous interpersonal thinking groups. Patients with more dichotomous interpersonal thinking had an earlier age at onset, higher self-certainty, lower estimation of their cognitive reserve (vocabulary subtest of WAIS), lower abilities for abstract thinking (similarities subtest of WAIS), poorer executive functioning (number of categories completed and perseverative errors in the WCST), and more severity of excitative symptoms than patients with low levels of dichotomous interpersonal thinking, in all cases with a moderate effect size. There were no statistically significant differences in the other sociodemographic and clinical factors.

**Table 1:** Comparison of two groups of patients with psychosis which were divided according to low and high levels of dichotomous interpersonal thinking.

	**Full sample (*N* = 85) %**	**Low DIT group(*n* = 42)%**	**High DIT group (*n* = 43) %**	**Statistical difference (χ^2^)**	**df**	***p***	**Cramer's V [95% CI]**
Gender (% males)	63.5	69.2	58.7	1.01	1	0.315	0.11 [0.108; 0.34]
Early psychosis	30.6	35.9	26.1	0.96	1	0.328	0.11 [0.106; 0.34]
Marital status (single)	72.9	69.2	76.1	6.43	4	0.169	0.275 [0.22; 0.49]
Secondary studies completed	45.0	41.0	47.8	7.85	5	0.164	0.304 [0.24; 0.51]
Incapacity for employment	37.6	25.6	47.8	5.78	5	0.328	0.261 [0.24; 0.47]
Diagnosis of schizophrenia	45.9	35.9	54.3	5.19	5	0.393	0.247 [0.24; 0.45]
	**Mean (SD); Range**	**Mean (SD)**	**Mean (SD)**	**Statistical difference (t)**	**df**	***p***	**Cohen's d [95% CI]**
**Socio-demographics**							
Age	37.1 (9.57); 19–57	37.67 (8.83)	36.54 (10.22)	0.537	83	0.593	0.12 [−0.31; 0.54]
Years of disorder	11.4 (8.78); 0.5–39	10.15 (8.35)	12.5 (9.09)	−1.230	83	0.222	−0.27 [−0.69; 0.16]
Age at onset	25.6 (7.54); 13–46	27.29 (7.30)	24.04 (7.50)	2.032	83	0.045	0.44 [0.07; 0.93]
Number of hospitalizations	3.20 (3.98); 0–22	2.82 (3.26)	3.52 (4.51)	−0.808	83	0.421	−0.18 [−0.6; 0,26]
Antipsychotic dosage^a^	28 (324); 0–2292	177.25 (171.15)	270.04 (408.6)	−1.305	81	0.195	−0.29 [−0.71; 0.14]
**Metacognition**							
BCIS self-reflectivity	14.7 (4.36); 4–27	14.49 (4.19)	14.96 (4.56)	−0.487	82	0.627	−0.11 [−0.53; 0.32]
BCIS self-certainty	8.07 (3.32); 1–18	7.19 (2.54)	8.95 (3.79)	−2.254	82	0.025	−0.54 [−0.97; −0.11]
**Neurocognition**							
WAIS vocabulary subtest	106 (13.1); 70–140	110.83 (12.24)	102.5 (12.67)	3.196	83	0.002	0.69 [0.25; 1.13]
WAIS similarities subtest	11.86 (3.25); 2–19	12.82 (3.22)	11.07 (3.09)	2.553	82	0.013	0.55 [0.12; 0.99]
WCST perseverative errors	42.5 (8.11); 29–57	44.52 (8.26)	40.67 (7.82)	2.205	81	0.030	0.48 [0.05; 0.91]
WCST categories	3.82 (2.11); 0–6	4.42 (1.97)	3.24 (2.1)	2.613	81	0.011	0.57 [0.13; 1.00]
**Symptomatology**							
PANSS excitative	5.19 (1.68); 4–11	4.83 (1.19)	5.52 (2.0)	−2.024	83	0.054	−0.43 [−0.87; −0.01]
PANSS cognitive	4.98 (1.91); 3–10	4.59 (1.71)	5.3 (2.03)	−1.735	83	0.086	−0.38 [−0.81; 0.05]
PANSS positive	7.39 (3.23); 4–16	6.77 (2.8)	7.91 (3.49)	−1.645	83	0.104	−0.36 [−0.79; 0.07]
PANSS depressive	11.8 (4.31); 5–23	11.26 (3.98)	12.2 (4.56)	−1.002	83	0.319	−0.22 [−0.64; 0.21]
PANSS expressive deficits	5.31 (2.64); 4–16	5.18 (2.02)	5.41 (3.08)	−0.404	83	0.687	−0.09 [−0.51; 0.34]
PANSS experiential deficits	5.87 (3.21); 3–15	5.79 (3.05)	5.93 (3.38)	−0.199	83	0.843	−0.04 [−0.47; 0.38]

a *Antipsychotic drug doses are expressed as chlorpromazine equivalence; DIT, Dichotomous interpersonal thinking; Early psychosis, 5 or under 5 years of evolution of the disease; PANSS, Positive and Negative Symptoms Scale; BCIS, Beck Cognitive Insight Scale; WCST, Wisconsin Card Sorting Test; WAIS, Wechsler Adult Intelligence Scale*.

Table 2 shows the hierarchical logistic regression analysis for predicting dichotomous interpersonal thinking with the variables that were found to associate with it. On a first analysis, we removed from our model the perseverative errors index of the WCST due to its high detected multicollinearity [variance inflation factor (VIF) of 4.14 and a detected correlation of 0.818 with the categories completed index of WCST], we based this selection on considering that the number of categories completed seemed to be a more adequate index for measuring cognitive flexibility (50). The other variables showed adequate levels of collinearity (VIFs all under 1.90 once this index was removed). In the first step, age at onset was entered, but it did not result on a significant model. In the second step, the metacognitive measure (self-certainty) was included, which resulted on a significant model that accounted for 6.6% (McFadden's R^2^) and 12% (Nagelkerke's R^2^) of the variance. The model comparison with respect to step 2 was also statistically significant. Finally, the final full model was composed of age at onset, self-certainty, and one domain of neurocognition, the estimated cognitive reserve (model of step 3). In the introduction of other domains of neurocognition, the similarities subtest of WAIS and of the WCST index of categories completed in subsequent steps did not add statistically significant contributions to the model (based on model comparisons). The final model showed adequate fit to the data and explained between 14% (McFadden's R^2^) and 24% (Nagelkerke's R^2^) of the variance, correctly classifying 67.9% of the cases, with a sensitivity capacity of 0.716 and a specificity of 0.641. As shown in Table 2, after the estimated cognitive reserve was added and according to the *p*-values, an earlier age at onset and a lower self-certainty in the model did not reach statistical significance to explain their classification as the high or low dichotomous interpersonal thinking groups. However, according to the odds ratio (effect size), the strongest predictor was self-certainty, followed by estimated cognitive reserve.

**Table 2:** Hierarchical logistic regression models predicting level of dichotomous interpersonal thinking.

**Predictor**	**AIC**	**Pseudo R^**2**^ (McFadden's–Nagelkerke's)**	**Model comparison (X^**2**^, *p*)**	**Overall model test (X^**2**^, *p*)**	**Omnibus likelihood ratio test (X^**2**^, *p*)**	**Log Odds ratio (*SE*)**	**Odds ratio**	**Odds ratio 95% *CI***
Step 1	113	0.028–0.052		3.19 (0.074)				
Constant						1.69 (0.82)	4,248	0.857–21.05
Age at onset					3.619(0.074)	−0.05 (0.03)	0,948	0.892–1.01
Step 2	110	0.066–0.12	4.84 (0.028)	8.03 (0.018)				
Constant						−0.05 (1.07)	0,947	0.115–7.77
Age at onset					2.03 (0.154)	−0.04 (0.03)	0,957	0.899–1.02
Self-certainty					4.84 (0.028)	0.16 (0.08)	1,172	1,011–1.36
Step 3	104	0.14–0.24	8.05 (0.005)	16.08 (0.001)				
Constant						6.31 (2.70)	554,031	4.39–221933.69
Age at onset					1.60 (0.206)	−0.04 (0.03)	0,959	0.898–1.024
Self-certainty					2.72 (0.099)	0.12 (0.08)	1,134	0.972–1.321
Estimated cognitive reserve					8.05 (0.005)	−0.06 (0.02)	0,944	0.904–0.988

*Odds represents the ratio of “High level of dichotomous interpersonal thinking” vs. “Low level of dichotomous interpersonal thinking;” AIC, Arkaike Information Criteria; SE, Standard error; CI, Confidence Interval*.

## Discussion

In this study, we tested differences in neurocognition and metacognition in patients with psychosis. To this aim, we compared individuals with high and low scores in dichotomous thinking. Our results show that the group with high dichotomous interpersonal thinking had poorer performance in self-certainty and executive functioning. This group also had an earlier age at onset, impaired abstract thinking, and lower estimated cognitive reserve than the group with flexible thinking. Finally, according to the logistic regression model, the factors that differentiated between the two groups were estimated cognitive reserve, followed by self-certainty.

Participants in the group with high interpersonal dichotomous thinking were limited in their cognitive flexibility. Also, their sense of self and perception of interpersonal relationships was characterized by cognitive rigidity as conceptualized from the neurocognitive (executive functioning) and metacognitive (self-certainty) domains. Additionally, these three constructs were measured using three different assessment approaches: a semi-structured interview, the RGT (dichotomous interpersonal thinking), a neuropsychological task (executive functioning), and a self-reported questionnaire (self-certainty). This convergence may support the idea that cognitive rigidity may be a generalized cognitive disruption present in some people with psychosis that manifests itself in specific domains such as neurocognition, metacognition, and the view of self and significant others. Patients in this group also showed earlier age at onset, a finding that is congruent with extensive research, suggesting that patients with earlier age at onset are more impaired in executive functioning and general cognitive abilities (51, 52). Our findings give further support to the idea that earlier age at onset could be a surrogate of disorder severity (53, 54), while high cognitive rigidity could be another marker of this severity.

Despite these results, the logistic regression showed that the domains that best differentiated patients with cognitive rigidity from those who with a more flexible, less polarized thinking pattern in the interpersonal context were the estimated cognitive reserve and the self-certainty index of cognitive insight. Although patients with high dichotomous interpersonal thinking had more impaired executive functioning as measured with the WCST, this measure did not contribute to explain the differences in the logistic regression model, which was an unexpected result. This result suggests that cognitive rigidity in the perception of self and others may rely more on basic cognitive abilities connected to cognitive reserve and on metacognitive processes related to overconfidence and rigidity to consider alternative explanations, rather than in specific abilities for flexibility in set-shifting. To the best of our knowledge, this is the first study to empirically support this idea. It has been suggested that overconfidence in own judgments may be influenced by acquired knowledge from past experience (55), which aligns with our results. Indeed, the inner construction of self and significant other is necessary built based on basic cognition, previous experiences, and metacognitive processes (22). It is not surprising that cognitive reserve emerged as a determinant factor, as in light of recent findings, patients with higher IQ are more likely to improve under metacognitive interventions (56). These results also support a growing body of evidence reporting that reasoning processes are underpinned by general cognitive functions (10, 35).

In a previous analysis, we found a small but significant association between high interpersonal rigidity and more positive symptoms (23). However, in the present study, we found that the severity of positive symptomatology did not differ between patients with higher and lower dichotomous interpersonal thinking. This result also contrasts with many other studies that related cognitive rigidity to increased positive symptoms (57). It could be that cognitive rigidity may be a cognitive bias related to but independent of the severity of positive symptoms, which could be influenced by many factors, thus being a stable trait of the disorder in a subgroup of patients. However, some considerations regarding our sample characteristics may also be considered. One explanation may be that we included patients with different symptom profiles of the psychotic spectrum, and the main literature in this topic has studied the presence of cognitive rigidity in active-deluded patients (2, 32).

Our study has some limitations that might affect the generalizability of the findings. First, the cross-sectional design of the study prevents drawing conclusions about causality; therefore, longitudinal studies are needed. Second, we conducted a short screening of neurocognitive and cognitive insight impairment. A more exhaustive assessment is needed to explore the links between dichotomous interpersonal thinking and comprehensive measures of cognitive biases and neurocognition. For instance, it is unknown whether other neurocognitive factors such as processing speed, working memory, or attention might have an influence on these results, as these factors have been shown to be affected in the presence of high self-certainty (6). A measure of belief inflexibility when reasoning about delusions should also be included in future studies to refine the validity of the study presented in this paper (5). Moreover, the relationship with other relevant cognitive biases that may share this underlying process of rigidity should be tested, such as need for closure, bias against disconfirmatory evidence, or jumping to conclusions (32, 58, 59). Third, regarding the sample characteristics and despite its clinical and functional heterogeneity, the proportion of chronic patients was much bigger than the proportion of recent-onset patients. Future studies with a focus on recent-onset patients would be needed. Finally, due to the bimodal distribution of the dichotomous interpersonal thinking index, we used the median split for dividing the groups. This statistical method for establishing groups has some advantages but has some drawbacks (48), so there may be other more complex statistical approaches that could yield different results. Different approaches to establishing groups according to their level of cognitive rigidity should be tested in future studies.

Despite the aforementioned limitations, our results may have implications for research and clinical practice. Chief among them is considering that cognitive rigidity may be a cognitive bias more generalized than previously considered that affects not only neurocognitive and metacognitive processes but also the sense of self and identity. Cognitive rigidity would be present in a subgroup of patients suffering psychosis. One possibility is that this subgroup of patients may benefit from decreasing their all-or-nothing tendency in their thinking pattern. To address this issue, the therapeutic work may be better approached by reducing the overconfidence in own judgments when thinking about themselves and the others. However, this intervention should always be adapted to the general intellectual level of each individual.

If these cognitive process rely on previous acquired knowledge and general cognitive abilities, they could be more amenable to change by using personalized interventions that focus on cognitive content (for example, cognitive-behavioral and metacognitive interventions) (55) and adapted to their unique interpersonal context. Alternatively, guaranteeing a minimum level of general cognitive abilities before considering intervention in cognitive rigidity may be recommended to maximize its benefit (56). Summarizing, patients showing high interpersonal dichotomous thinking might benefit from interventions that target this cognitive bias on an integrative way and adapted to their general level of intelligence. These suggestions should be tested in clinical trials of cognitive behavioral and metacognitive interventions in which changes in cognitive rigidity occur (31, 60, 61) or are expected to mediate the improvement in psychotic symptomatology (10, 27).

## Data Availability Statement

The raw data supporting the conclusions of this article will be made available by the authors, without undue reservation.

## Ethics Statement

The studies involving human participants were reviewed and approved by Research Ethics Committee of Parc Sanitari Sant Joan de Déu. The patients/participants provided their written informed consent to participate in this study.

## Author Contributions

HG-M, SO, and GF designed the study and interpreted the results. HG-M wrote the first draft of the manuscript, collected the data for the study, and ran the statistical analyses. JU collaborated with the collection of data. HG-M, JU, GF, and SO edited the first draft of the manuscript. All authors approved the final version of the manuscript.

## Conflict of Interest

The authors declare that the research was conducted in the absence of any commercial or financial relationships that could be construed as a potential conflict of interest.
